# Treatment of microvascular angina pectoris by activating blood circulation to remove blood stasis: A systematic review and meta-analysis

**DOI:** 10.1097/MD.0000000000040012

**Published:** 2024-10-11

**Authors:** Ruitong Yang, Zelin Bai, Chao Liang, Feng Qi

**Affiliations:** a School of Traditional Chinese Medicine, Changchun University of Chinese Medicine, Changchun, China; b Hospital of Affiliated Changchun University of Chinese Medicine, Changchun, China.

**Keywords:** activate blood circulation and remove blood stasis, clinical efficacy, meta-analysis, MVA

## Abstract

**Background:**

To systematically evaluate the clinical efficacy of using the method of activating blood circulation and removing blood stasis in the treatment of microvascular angina (MVA) based on the meta-analysis method.

**Methods:**

A comprehensive search of randomized controlled trials on the treatment of MVA using the method of activating blood circulation and removing blood stasis in Chinese and English databases such as China National Knowledge Infrastructure Database, Wanfang Data Knowledge Service Platform, Vipshop Journal Resource Integration Service Platform, PubMed, Cochrane Library, Embase, Web of Science, and CBM was conducted, and the search time limit was from the establishment of each database to February 2023. All retrieved literature was screened, and data extracted according to the specified inclusion and exclusion criteria, and data analysis was completed using Revman 5.4 software.

**Results:**

Fifteen studies with a total of 1031 patients were finally included, and the results of the meta-analysis showed that the treatment with the method of activating blood circulation and removing blood stasis was more advantageous compared with the group of Western medicine alone. Among them, clinical effect of activating blood circulation and removing blood stasis (relative risk = 1.20, 95% confidence intervals [CI] [1.11, 1.31], *P* < .0001) and electrocardiographic efficacy (relative risk = 1.32, 95% CI [1.15, 1.51], *P* < .0001) were more effective, concentration levels of endothelin-1 (standardized mean difference = −2.14, 95% CI [−2.97, −1.31], *P* < .00001) and high sensitivity C-reactive protein (mean difference = −0.84, 95% CI [−0.93, −0.75], *P* < .00001) were lower, concentration levels of nitric oxide (standardized mean difference = 0.61, 95% CI [0.33, 0.89], *P* < .0001) was higher, and ST-segment depression range (mean difference = −0.07, 95% CI [−0.09, −0. 04], *P* < .00001) was smaller.

**Conclusion:**

Compared with the treatment with Western medicine alone, the treatment of MVA is more effective by choosing the method of activating blood circulation and removing blood stasis.

## 1. Introduction

Microvascular angina (MVA), also known as cardiac syndrome X, has typical angina symptoms with ST-segment depression or other objective basis of myocardial ischemia, normal coronary angiography and exclusion of other diseases causing angina symptoms.^[1]^ In recent years, with the development of evidence-based medicine and the maturity of coronary revascularization, coronary microcirculation dysfunction plays an important role in ischemic cardiomyopathy. The coronary microcirculation system is composed of the coronary arterioles (diameter < 300 μm), capillary (diameter 5–8 μm), and venules (diameter < 500 μm). Several studies have shown that the incidence of cardiovascular events and mortality in patients with MVA increases yearly.

The pathophysiologic mechanism of MVA has not been clearly defined and may be related to factors such as coronary microvascular endothelial cell dysfunction, inflammatory response, coronary atherosclerosis, blood rheology and lipid metabolism.^[2]^ Some studies have suggested that besides traditional cardiovascular risk factors, some nontraditional risk factors of coronary heart disease, such as serum endothelin-1 (ET-1), nitric oxide (NO), high sensitivity C-reactive protein (hs-CRP), and other inflammatory factors are also associated with MVA.^[3]^ Standard treatment uses nitrates, β-blockers and statins to increase myocardial blood supply, reduce myocardial oxygen consumption and protect vascular endothelial cells from inflammatory damage. Through standard treatment can regulate blood lipids, reduce blood viscosity, inhibit platelet aggregation and anti-inflammation, so as to promote smooth channel, reduce cardiovascular risk and improve prognosis. According to the traditional Chinese medicine, the cause of MVA is the deficiency of Qi in upper Jiao, the exuberance of pathogenic Qi in lower Jiao, and the deficiency of Yang-Qi in upper Jiao, which leads to the upward invasion of cold pathogenic Qi in lower Jiao, leading to the exchange of the 2 positions, cold pathogen is mainly stagnation and absorption, so the upper Jiao-Yang Qi is blocked, the blood cannot run normally, and finally cause blood stasis in the blood vessels, resulting in chest obstruction and heartache. In order to further investigate the clinical efficacy of the method of activating blood circulation and removing blood stasis in the treatment of MVA, this study analyzed and discussed the adopted literature using a systematic evaluation method, so as to provide more evidence-based basis for the treatment method of activating blood circulation and removing blood stasis. This study was registered with the International Registry for prospective systematic reviews, registration number CRD42024473945.

## 2. Materials and methods

### 2.1. Eligibility criteria

#### 2.1.1. Inclusion criteria

(1) The study subjects were patients who had been clearly diagnosed with MVA, and other diseases caused by chest pain were excluded, and no other organic diseases were combined. The course of disease, gender and age were not limited for the cases. (2) Interventions: The experimental group was given standard treatment combined with blood-vitalizing and blood-stasis-removing drugs, and the control group was given standard treatment. Standard treatment included vasodilators, β-blockers, statins, etc. (3) The trial design was a randomized controlled study with no restriction on whether the included studies were blinded. (4) The outcome indicators were clinical efficacy, electrocardiographic (ECG) efficacy, ET-1, NO, hs-CRP, and ST-segment depression amplitude.

#### 2.1.2. Exclusion criteria

(1) Duplicate publications or literature with incomplete data information. (2) Nonclinical trial studies of reviews, animal experiments or drug studies. (3) Non-randomized controlled studies. (4) Studies related to the addition of drugs other than blood-activating and stasis-transforming drugs to interventions in the control group or the use of non-pharmacological treatments (e.g., acupuncture, acupressure, and extracorporeal counterpulsation) in the trial group.

### 2.2. Information sources and search strategy

We searched Chinese and English databases such as China National Knowledge Infrastructure Database, Wanfang Data Knowledge Service Platform, Vipshop Journal Resource Integration Service Platform, PubMed, Cochrane Library, Embase, Web of Science and CBM. The search time frame was from the establishment of each database to February 2023. The following keywords were utilized in the literature search strategy: “microvascular angina pectoris,” “Cardiac X syndrome,” “cardiac syndrome X,” “Coronary Microvascular Disease,” “Coronary microvascular dysfunction,” “Angina with normal coronary arteries,” “Microvascular dysfunction,” “Coronary microvascular disease,” “Coronary small vessel disease,” and “treatment.” Synonymous Chinese substitutions were used in the search of Chinese databases.

### 2.3. Selection process and data collection process

Two researchers were selected to each screen all retrieved literature, exclude incompatible literature according to the literature inclusion criteria and extract general data from the final included literature, including the name of the first author, time of publication, number of included cases, basic patient information, interventions, and outcome indicators. In case of disagreement between the two individuals, the study was referred to a third party for discussion.

### 2.4. Study risk of bias assessment

Risk of bias evaluation of included studies was performed using Cochrane Risk of Bias tool (RoB 2.0), and methodological quality evaluation of randomized controlled clinical trials, which included: generation of randomized sequences; allocation concealment; blinding of investigators and subjects; blinded evaluation of study outcomes; completeness of literature outcome data; and presence of bias. The evaluation was referred to 2 investigators independently, and in case of disagreement between them, the study was referred to a third party for discussion.

### 2.5. Synthesis methods

Meta-analysis was performed using RevMan 5.4. Relative risk (RR) was selected for analysis for dichotomous variables and standardized mean difference (SMD) or mean difference (MD) for continuous variables, and 95% confidence intervals (CI) were calculated for both. Heterogeneity tests were performed on the screened literature, and if the studies were homogeneous (*P* ≥ .1, I2 ≤ 50%), a fixed-effects model was used to analyze the data; if the studies were more heterogeneous (*P* < .1, I2 > 50%), sensitivity analysis or subgroup analysis was performed to further analyze the sources of heterogeneity, and a random-effects model was used to analyze the data. Inverted funnel plot analysis was used to assess publication bias.

## 3. Results

### 3.1. Study selection

**Figure 1.**
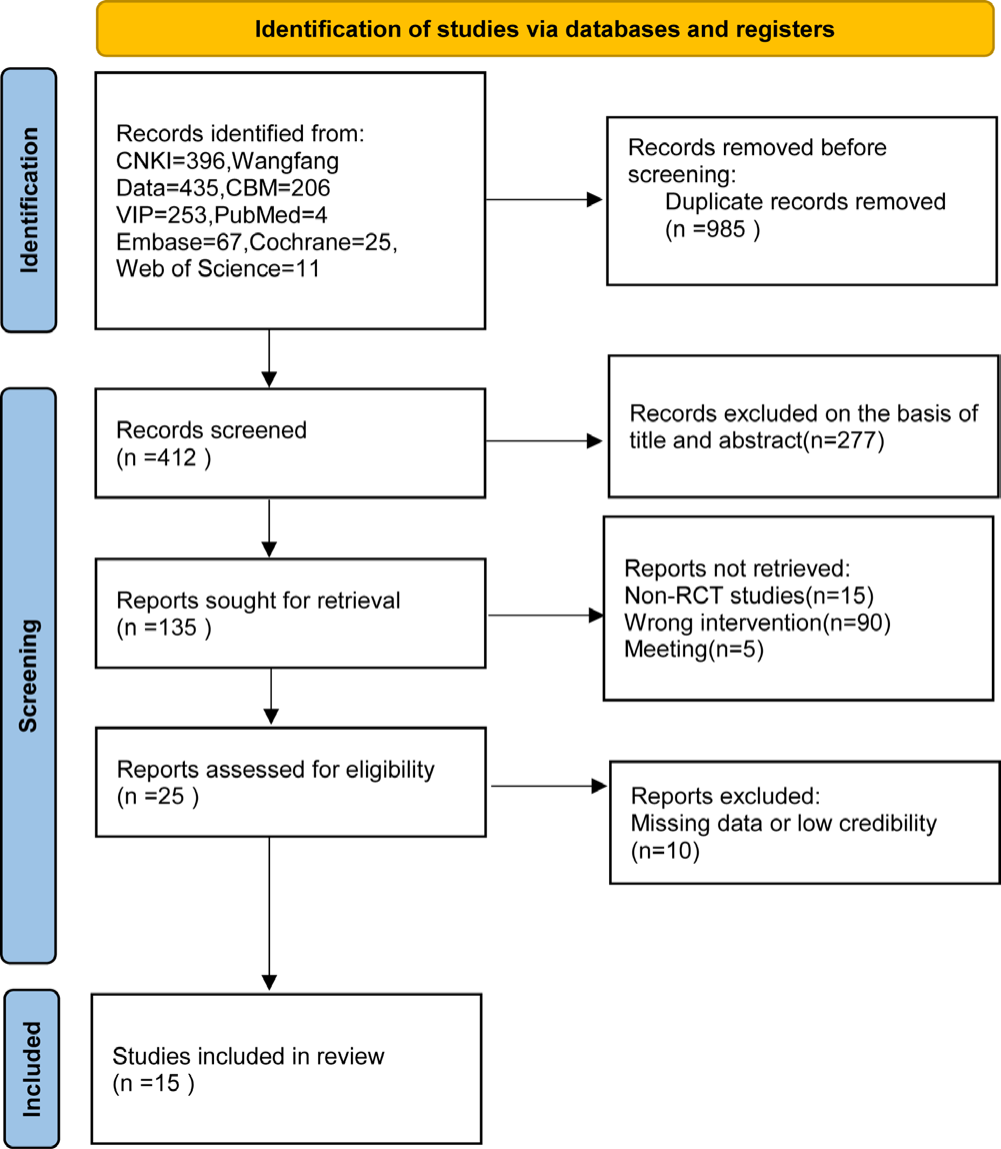
Flow chart of literature screening of blood activation and blood stasis treatment for MVA. MVA = microvascular angina.

In this study, a total of 1397 relevant papers were retrieved by computer, including 396 papers in China National Knowledge Infrastructure Database, 435 papers in WanFang Data, 253 papers in Vip database, 206 papers in CBM, 4 papers in PubMed, 11 papers in Web of Science, 67 papers in Embase, and 25 papers in Cochrane Library. And 15 papers were finally included after the initial screening and rescreening by excluding duplicate papers. Figure 1 shows the specific process and results of the screening.

### 3.2. Study characteristics and results of individual studies

A total of 15 papers were included in this study, which involved a total of 1031 cases, 537 in the test group and 494 in the control group. Table 1 shows the basic characteristics of the included literature.

**Table 1:** Basic characteristics of the studies included in the treatment of microvascular angina by activating blood circulation and removing blood stasis.

Study	Year	Sample size/case		Sex (male/female)	Age (*x* ± *s*, year)	Interventions		Treatment duration	Ending indicators
		Test group	Control group			T	C		
Liang Chun^[4]^	2007	21	21	T:7/14 C:5/16	T:55.1 ± 3.9 C:56.7 ± 3.4	R + Danshen tablet(3 pills)	Regular + Danshen simulated pills	4 wk	①④⑤
Xiong Lu^[5]^	2019	60	60	T:21/39 C:19/41	T:51.2 ± 8.7 C:50.8 ± 7.9	R + Ginkgo Biloba oral liquid (10 mL)	Regular	12 wk	①②③⑤
Fu Yongyong^[6]^	2020	32	32	T:12/20 C:10/22	T:44.8 ± 7.3 C:45.5 ± 6.7	R + Kedaling tablet (3 pills)	Regular	12 wk	①⑤
Chen Huijun^[7]^	2021	30	30	T:10/20 C:14/16	T:54.265 ± .78 C:56.29 ± 6.01	R + Danshen Yin (300 mL)	Regular	4 wk	①
Chen Bin^[8]^	2018	47	45	T:22/25 C:21/24	T:44.2 ± 12.2 C:43.1 ± 12.5	R + Danhong injection (40 mL)	Regular	2 wk	①④
Xu Lingjian^[9]^	2002	36	20	T:23/13 C:13/7	T:38 ± 6.7 C:37 ± 9.3	R + Shuanshi capsule (3 pills)	Regular	1 wk	③
Jiang Zhaolin^[10]^	2021	43	43	T:28/15 C:27/16	T:47.87 ± 5.42 C:47.98 ± 5.41	R + Fufangwenban decoction (100 mL)	Regular	1 mo	①②③④
Wu Lihua^[11]^	2010	26	24	T:10/16 C:9/15	T:44.34 ± 4.41 C:44.42 ± 5.05	R + Shuxuening injection (20 mg)	Regular	15 d	①④⑤
Li Long^[12]^	2010	30	30	T:14/16 C:15/15	T:45.5 ± 4.5 C:44.28 ± 5.72	R + Shuxuetong injection (6 mL)	Regular	3 wk	①⑤
Li Long^[13]^	2017	30	30	T:17/13 C:16/14	T:61.4 ± 8.0 C:62.4 ± 8.5	R + Xinxuean granule (1 bag)	Regular	28 d	①
Ji Yingmin^[14]^	2009	45	31	T:26/19 C:22/9	T:55.8 ± 8.1 C:53.7 ± 7.7	R + Shuxuetong injection (6 mL)	Regular + Isosorbide mononitrate 20 mg	2 wk	①⑤
Qiu Zhichao^[15]^	2021	40	40	T:32/8 C:31/9	T:57 ± 11 C:60 ± 9	R + Ginkgo Biloba oral liquid (10 mL)	Regular	2 mo	①⑤
Zu Xiaolin^[16]^	2012	35	35	T:15/20 C:12/23	T:59.8 ± 10.3 C:62.4 ± 11.5	R + Sulfotanshinone Sodium injection (80 mg)	Regular	2 wk	①③④
Sun Mingqiang^[17]^	2012	32	33	T:13/19 C:14/19	T:50.34 ± 3.84 C:51.93 ± 3.93	R + Ginkgo leaf extract and Dipyridamole injection (20 mL)	Regular	15 d	①⑤
Niu Tianfu^[18]^	2008	30	20	T:13/17 C:8/12	T:52 ± 3 C:53 ± 2	R + Maixuekang capsule (4 pills)	Regular	1 mo	①⑤

T = test group, C = control group. Ending indicators: ① Clinical efficacy; ② NO; ③ ET-1; ④ hs-CRP; ⑤ ST-segment depression amplitude.

### 3.3. Risk of bias in studies

**Figure 2.**
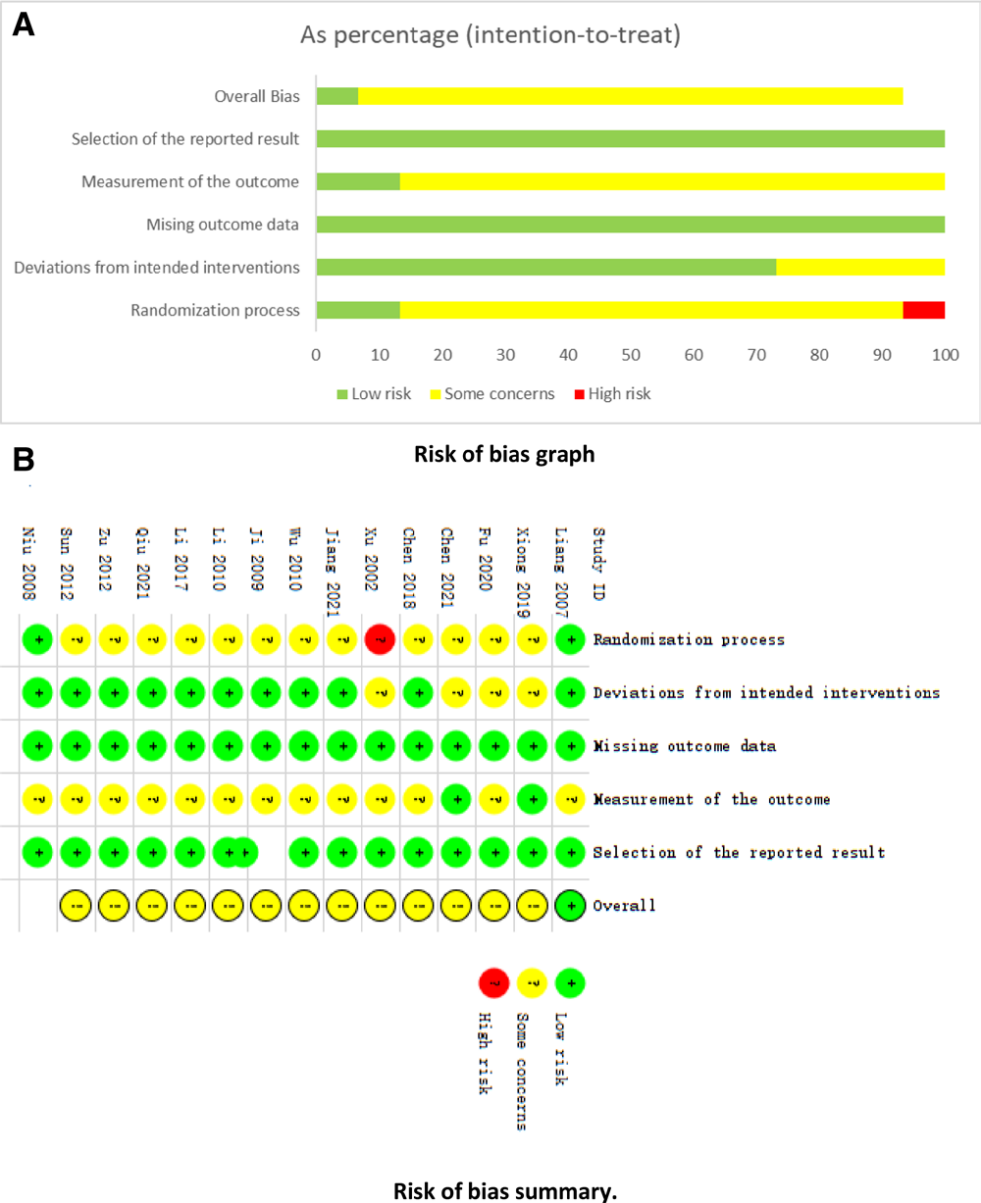
Inclusion of study bias in the treatment of MVA by activating blood circulation and removing blood stasis. MVA = microvascular angina.

Fourteen of the fifteen included papers^[4–8,10–18]^ mentioned randomized grouping, of which 4 papers^[5,6,10,15]^ were grouped using the random number table method; 2 papers^[4,14]^ reported blinding; none of the selected papers reported allocation concealment; all studies reported complete outcome indicator results; and no other bias was explicitly mentioned in all papers. The specifics of the quality assessment of the included studies can be seen in Figure 2.

### 3.4. Results of syntheses

#### 3.4.1. Clinical efficacy

**Figure 3.**
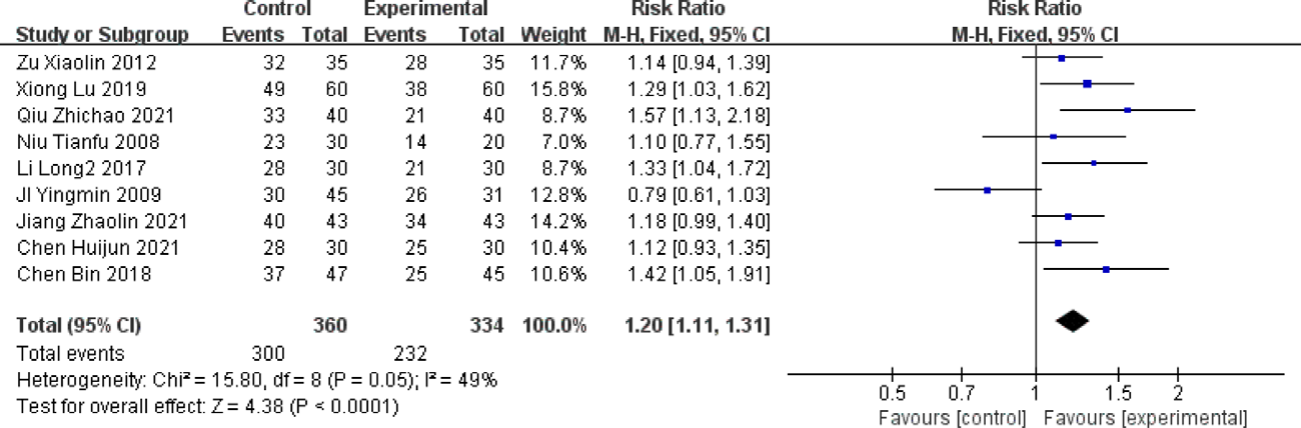
Forest plot of clinical efficacy of blood activation and blood stasis treatment for MVA. MVA = microvascular angina.

Of the 15 included papers, 9 papers reported angina outcome indicators,^[5,7,8,10,13–16,18]^ and the studies were homogeneous (*P* = .06, I2 = 46%), so meta-analysis was performed using a fixed-effects model, angina pectoris efficacy combined with effect size (RR = 2.40, 95% CI [1.66, 3.46], *P* < .0001), the results showed that the treatment of angina pectoris in MVA with the method of activating blood circulation and removing blood stasis was more effective compared with the treatment with Western medicine alone. For details, see Figure 3.

#### 3.4.2. ECG efficacy

**Figure 4.**
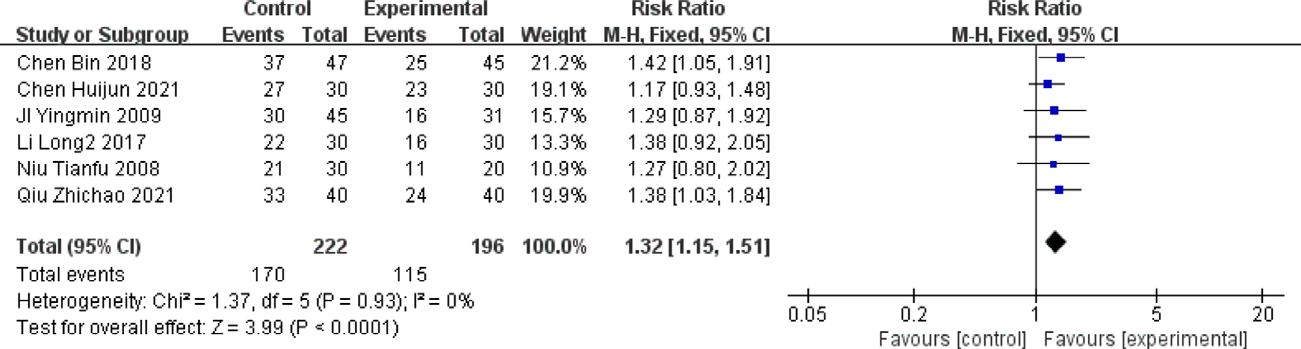
Forest plot of ECG efficacy of blood activation and blood stasis treatment for MVA. ECG = electrocardiographic, MVA = microvascular angina.

Of the 15 included papers, 6 papers reported ECG efficacy outcome indicators,^[7,8,13–15,18]^ and the studies were homogeneous (*P* = .93, I2 = 0%), so meta-analysis was performed using a fixed-effects model, ECG efficacy combined with effect size (RR = 1.32, 95% CI [1.15, 1.51], *P* < .0001). The results showed that the ECG efficacy of MVA was better with the choice of blood activation and blood stasis treatment compared with western medicine treatment alone. For details, see Figure 4.

#### 3.4.3. Serum ET-1

**Figure 5.**
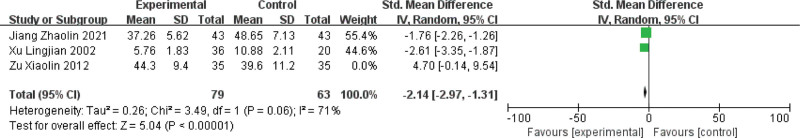
Forest plot of serum vascular ET-1 level by activating blood circulation and removing blood stasis in the treatment of MVA. ET-1 = endothelin-1, MVA = microvascular angina.

Among the 15 included papers, 3 papers reported serum ET-1 levels,^[9,10,16]^ and the heterogeneity among studies was large (*P* < .00001, I2 = 97%). Sensitivity analysis was performed on the original literature (some papers were excluded one by one and analyzed again), considering the possible correlation with experimental methods, measuring instruments and treatment time, and after excluding the study by Zu Xiaolin,^[16]^ the I2 value fell back to 71%, *P* = .06. Therefore, a random-effects model was used for meta-analysis, and subgroup analysis was not performed because of the limited sample size. The results showed that ET-1 levels decreased in the experimental group after treatment, and the difference was statistically significant (SMD = −2.14, 95% CI [−2.97, −1.31], *P* < .00001), which indicates that the treatment with the method of activating blood circulation and removing blood stasis can effectively reduce serum ET-1 levels and thus improve vascular endothelial function compared with the treatment with Western medicine alone. For details, see Figure 5.

#### 3.4.4. Serum NO

**Figure 6.**
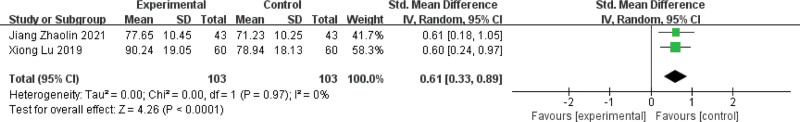
Forest plot of serum NO level by activating blood circulation and removing blood stasis method for MVA. MVA = microvascular angina, NO = nitric oxide.

Of the 15 included papers, 2 papers reported serum NO levels,^[5,10]^ and there was no heterogeneity among studies (*P* = .97, I2 = 0%), so meta-analysis using fixed-effects model showed that NO levels increased in the experimental group after treatment, and the difference was statistically significant (SMD = 0.61, 95% CI [0.33, 0.89], *P* < .0001), which indicates that the treatment with the method of activating blood circulation and removing blood stasis can effectively increase the serum NO level and thus improve the vascular endothelial function compared with the treatment with Western medicine alone. For details, see Figure 6.

#### 3.4.5. Serum hs-CRP

**Figure 7.**
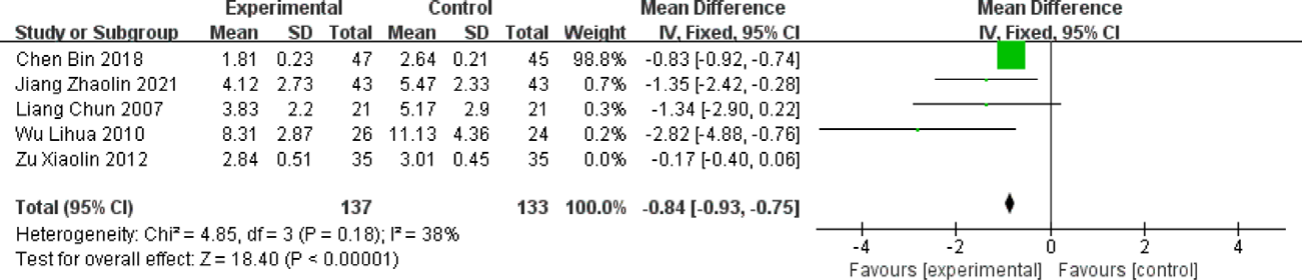
Forest plot of serum ultrasensitive C-reactive protein level by activating blood circulation and removing blood stasis method for MVA. MVA = microvascular angina.

Of the 15 included papers, 5 papers reported serum hs-CRP levels,^[4,8,10,11,16]^ and the heterogeneity of the studies was known to be large by combined analysis (*P* < .00001, I2 = 89%), sensitivity analysis was performed (some papers were excluded in turn and the analysis was repeated), and after excluding the study by Zu Xiaolin,^[16]^ the I2 value fell back to 38%, *P* = .18, and each independent group was combined, so meta-analysis was performed using a fixed-effect model, and the results showed that the hs-CRP level decreased after treatment in the test group, and the difference was statistically significant (MD = −0.84, 95% CI [−0.93, −0.75], *P* < .00001), so it can be seen that, compared with the treatment of Western medicine alone, the treatment with the method of activating blood circulation and removing blood stasis can effectively reduce the serum hs-CRP level and thus improve the vascular endothelial function. For details, see Figure 7.

#### 3.4.6. ST-segment depression amplitude

**Figure 8.**
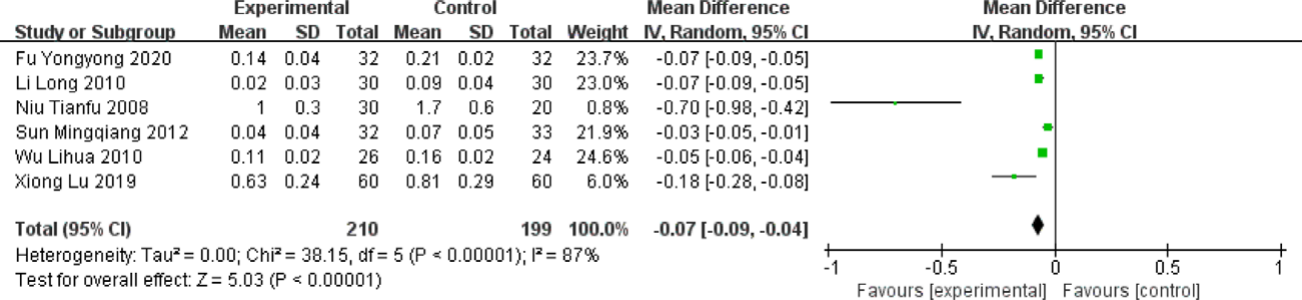
Forest plot of ST-segment depression amplitude in MVA by activating blood circulation and removing blood stasis. MVA = microvascular angina.

Among the 15 included papers, 6 papers reported ST-segment depression amplitude,^[5,6,11,12,17,18]^ with a large heterogeneity among studies (*P* < .00001, I2 = 87%). Sensitivity analysis of the original literature (excluding some papers one by one and analyzing again) did not reveal heterogeneous literature, so a random-effects model was used for the combined analysis. The results showed that the amplitude of the ST-segment depression decreased after treatment in the trial group, and the difference was statistically significant (MD = −0.07, 95% CI [−0.09, −0. 04], *P* < .00001), which showed that the treatment with blood activation and blood stasis can effectively reduce the amplitude of ST-segment depression and thus relieve angina pectoris symptoms compared with the treatment with Western medicine alone. For details, see Figure 8.

**Figure 9.**
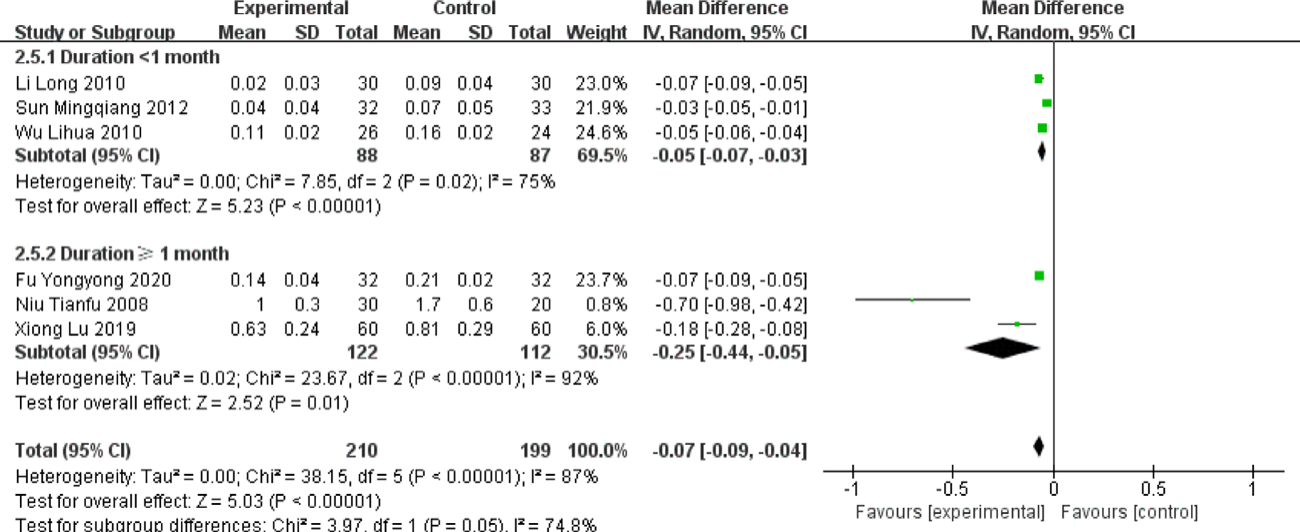
Forest plot of subgroup analysis of ST-segment depression amplitude for MVA by activating blood circulation and removing blood stasis. MVA = microvascular angina.

A subgroup analysis was performed on 6 papers, divided into 2 subgroups according to the duration of treatment (duration < 1 month, duration ≥ 1 month). The results showed that the test group in both subgroups was more effective in reducing the amplitude of ST-segment depression compared with the control group, and the difference was statistically significant (duration < 1 month: MD = −0.05, 95% CI [−0.07, −0.03], *P* < .00001; duration ≥ 1 month: MD = −0.25, 95% CI [−0.44, −0.05], *P* = .01). See Figure 9 for details.

### 3.5. Publication bias analysis

Funnel plotting analysis was not performed because the volume of literature reporting clinical efficacy was <10. The results of the included clinical efficacy studies for angina were not negative, from which it can be further speculated that there may be publication bias.

## 4. Discussion

This study included 15 studies on the treatment of MVA with the method of promoting blood circulation to remove blood stasis. The results showed that the treatment of MVA with the method of promoting blood circulation to remove blood stasis could further improve the clinical curative effect and ECG curative effect on the basis of the routine treatment of Western medicine, it increased NO secretion, decreased the release of ET-1 and hs-CRP, and relieved the depression of ST. These results suggest that the therapy of promoting blood circulation and removing blood stasis can effectively treat MVA, so as to relieve the symptoms of microcirculation and improve the quality of life of patients. As an independent predictor of cardiovascular events, coronary microcirculatory dysfunction plays a crucial role in MVA. The coronary circulatory system does not deliver oxygen and blood to the heart. When the circulation is impaired, myocardial ischemia and hypoperfusion follow, which leads to intravascular occlusion and spasm, resulting in irreversible damage to the myocardium. This is thought to be a pathological change of microcirculation. Niu Tianfu, Xiong Lu et al^[5,18]^ have confirmed through the traditional Chinese medicine improvement myocardial ischemia, realizes the myocardial microcirculation reperfusion, enhances the clinical curative effect.

Vascular endothelial function, as a first-line index to assess cardiovascular function, can release serum NO and serum ET-1, which modulate vascular contractility and diastolic capacity through these diastolic factors to maintain myocardial blood supply and oxygen balance, regulate the function of blood vessel wall. If the balance of endothelial secretion is broken, the systolic rhythm of coronary artery is changed, and the blood flow is abnormal, which leads to the accumulation of myocardial metabolite, and finally leads to the dysfunction of vascular endothelium, which causes the diseases such as MVA. Palmer^[19]^ demonstrated the role of NO in regulating myocardial contractility, improving blood flow and reducing the adhesion of vascular metabolites. Pan et al^[20]^ showed that tanshinone IIA protects vascular endothelial function by increasing NO concentration and inhibiting ET-1 production through NO synthase.

Inflammation is one of the predisposing factors of cardiovascular diseases. The invasion of pathogens leads to a series of proinflammatory cytokine, and the activation of inflammation eventually leads to the damage of the vessel wall and abnormal dilation of the coronary microvessels, insufficient blood flow. The study shows that the increase of hs-CRP can reflect the degree of vascular injury during ischemia-reperfusion, which provides a certain direction for clinical diagnosis and treatment. Chen bin, Liang Chun, et al^[4,8]^ found that Danhong injection and Danshen tablets can inhibit inflammatory factors, decrease hs-CRP and improve ST-segment ischemic depression to continuously improve the quality of life in MVA patients.

The heterogeneity of the outcome indicators in this study reflects the limitations of the findings in the literature, with the main problems being: the randomization method of the study was not stated, and whether blinding was used with allocation concealment was not mentioned; the sample size included in the study was insufficient and the length of treatment varied; long-term follow-up and adverse effects were not reported, and safety evaluation was not performed. Meta-analysis synthesized the results of many independent studies. Due to the difference of medical skill, dosage and quantity of drugs, it is impossible to be absolutely consistent between the included clinical research literature. Blinding is rarely discussed in the included literature, and only a few studies mention blinding, but do not detail its implementation, which will affect the quality of the literature. Large-scale, high-quality randomized double-blind trials are still needed to verify this. Evidence-based medicine design needs to be more rigorous, and researchers need to standardize clinical research, so as to increase the confidence of scholars in the results of clinical efficacy research.

## 5. Conclusions

In this study, we obtained the evidence-based evidence of the effectiveness of Chinese herbs for promoting blood circulation and removing blood stasis in the treatment of MVA patients through systematic review. Chinese herbs for promoting blood circulation and removing blood stasis could improve the symptoms of chest tightness and pain, and increase the secretion of NO in serum, by reducing the release of ET-1 and hs-CRP in serum, myocardial ischemia can be alleviated so as to reduce cardiovascular events and continuously improve patients’ quality of life, thus making traditional Chinese medicine get a broader development space. However, the present conclusions are based on a limited data set. To provide a robust validation of these findings, future studies should involve larger sample sizes and higher quality studies.

## Author contributions

**Conceptualization:** Chao Liang.

**Data curation:** Zelin Bai.

**Formal analysis:** Ruitong Yang.

**Funding acquisition:** Chao Liang.

**Investigation:** Chao Liang.

**Methodology:** Ruitong Yang.

**Project administration:** Chao Liang.

**Resources:** Zelin Bai.

**Software:** Zelin Bai.

**Supervision:** Feng Qi.

**Validation:** Chao Liang.

**Visualization:** Chao Liang.

**Writing – original draft:** Ruitong Yang.

**Writing – review & editing:** Ruitong Yang.
